# Behavioral and human-computer interaction requirements for a candidate abdominal-sound-enabled dietary self-regulation system: a two-phase study in Taiwanese university students

**DOI:** 10.3389/fnut.2026.1844650

**Published:** 2026-08-13

**Authors:** Tung-Jing Fang, Chuan Chia Wang, Jwo-Shiun Sun, Chen Han

**Affiliations:** 1 Department of Physiology and Biophysics, Graduate Institute of Physiology, College of Medicine, National Defense Medical University, Taipei, Taiwan; 2 Department of Electronic Engineering, National Taipei University of Technology, Taipei, Taiwan; 3 Doctoral Program in Design, College of Design, National Taipei University of Technology, Taipei, Taiwan

**Keywords:** abdominal sounds, digital nutrition, positive eating, human-computer interaction, wearable biofeedback, dynamic Kano model, Taiwanese university students

## Abstract

**Background:**

Wearable nutrition tools frequently prioritize calorie surveillance and retrospective logging. However, university students may require low-friction support that aligns with everyday eating contexts. The hypothesis that abdominal sounds could serve as biologically plausible future gastrointestinal timing cues is well-founded. The purpose of this study was to define the behavioral and human-computer interaction (HCI) requirements for a candidate abdominal-sound-enabled dietary self-regulation system among Taiwanese university students.

**Methods:**

The manuscript integrates two empirically linked phases. Phase 1 was a formative cross-sectional study of Taiwanese university students (*n* = 280; 45% male; mean age 22 ± 4 years) examining the Positive Eating Scale and its associations with eating behavior, diet quality, and BMI. Phase 2 was a randomized, formative 12-week intervention with 1:1 allocation (*n* = 200; *n* = 100 per group). A dynamic Kano analysis was employed to describe the evolution of HCI requirements over 12 weeks.

**Results:**

The Positive Eating Scale exhibited a two-factor structure (KMO = 0.85; Bartlett's chi-square (28) = 14,605.46, *p* < 0.001), demonstrating satisfactory reliability for satisfaction (alpha = 0.85), pleasure (alpha = 0.92), and the total score (alpha = 0.87). The results indicated that satisfaction, rather than pleasure, exhibited a more favorable behavioral profile, characterized by lower restrained eating (*r* = −0.38), higher intuitive eating (*r* = 0.49), better perceived health (*r* = 0.30), and lower BMI (*r* = −0.25). At the group level, outcomes favored the intervention group for fruit-and-vegetable intake, unhealthy snack frequency, device adherence, and user satisfaction; these findings are descriptive and should not be interpreted as adjusted estimates of efficacy. The standardized mean differences calculated from the reported means and standard deviations were substantial. Initially, attractive features migrated to one-dimensional attributes, whereas several one-dimensional features became must-be requirements.

**Conclusions:**

The extant evidence supports the implementation of a non-punitive, satisfaction-centered, adaptive, and low-friction interface architecture for future wearable dietary self-regulation. Abdominal sounds should be interpreted solely as a candidate timing/context input until participant-level acoustic validation and downstream gut-brain-axis endpoints are obtained.

## 1 Introduction

Dietary behavior is among the most modifiable determinants of metabolic and brain health (1, 2). Concurrently, the integration of personalized nutrition and consumer wearables has enabled the delivery of dietary support tailored to the individual's everyday physiology and context (3, 4). However, numerous nutrition technologies continue to operationalize diet as a surveillance task dominated by calorie counting, logging burdens, and static feedback (5, 6). This has the potential to reduce perceived usefulness and undermine long-term engagement (7, 8).

This challenge is especially salient in university students, whose eating behavior is shaped by irregular schedules, convenience-driven food environments, academic stress, social eating, and fluctuating self-regulatory capacity (9–11). In such contexts, the discrepancy between nutrition knowledge and real-world dietary behavior is not merely an information problem; it is also a design problem. The effectiveness of a system depends on its alignment with the user's rhythms, decision points, and tolerances for friction.

The present study is predicated on a central behavioral premise that satisfaction with eating and pleasure when eating should not be treated as interchangeable constructs. The Positive Eating Scale differentiates between a more extensive evaluative dimension of eating satisfaction and a more immediate hedonic dimension of eating pleasure (12). This distinction is of significance for the design of interventions. Research on intuitive eating and internal-cue-based regulation suggests that healthier eating patterns are supported not merely by maximizing enjoyment, but by building acceptance, bodily congruence, and sustainable self-regulation (13, 14).

The HCI literature offers two complementary frameworks for translating behavioral signals into design priorities. The technology acceptance model (TAM) emphasizes perceived usefulness and perceived ease of use as the primary determinants of adoption (5, 6). Conversely, just-in-time interventions and usability research suggest that timing, burden, and contextual relevance are pivotal for sustained engagement (7, 8). The Kano theory introduces a temporal dimension by distinguishing between must-be, one-dimensional, attractive, and indifferent attributes (15, 16). Dynamic extensions demonstrate that these categories migrate in response to exposure and expectation (17, 18). In the context of wearable nutrition systems, it is imperative to recognize that their novelty is unlikely to sustain value unless they evolve into reliable, integral components of daily life.

A secondary translational premise is biological plausibility. The diet–microbiota–gut–brain axis is increasingly recognized as a pathway through which dietary patterns influence cognition, mood, stress reactivity, and broader mental health outcomes (19–24). Nevertheless, a considerable number of studies examining the relationship between the gut and the brain continue to rely on limited measurements or on laborious laboratory endpoints. Abdominal sounds are therefore of interest as a candidate non-invasive and ecologically deployable signal of gastrointestinal activity. Recent studies have shown that bowel sound monitoring can be achieved in portable, wearable formats, with the potential to capture meal- or disease-related gastrointestinal patterns (25–28). It is important to note that such signals are not substitutes for microbiome sequencing, metabolomics, or neurocognitive testing. However, they may provide a practical timing layer for behavioral feedback.

**Figure 1:**
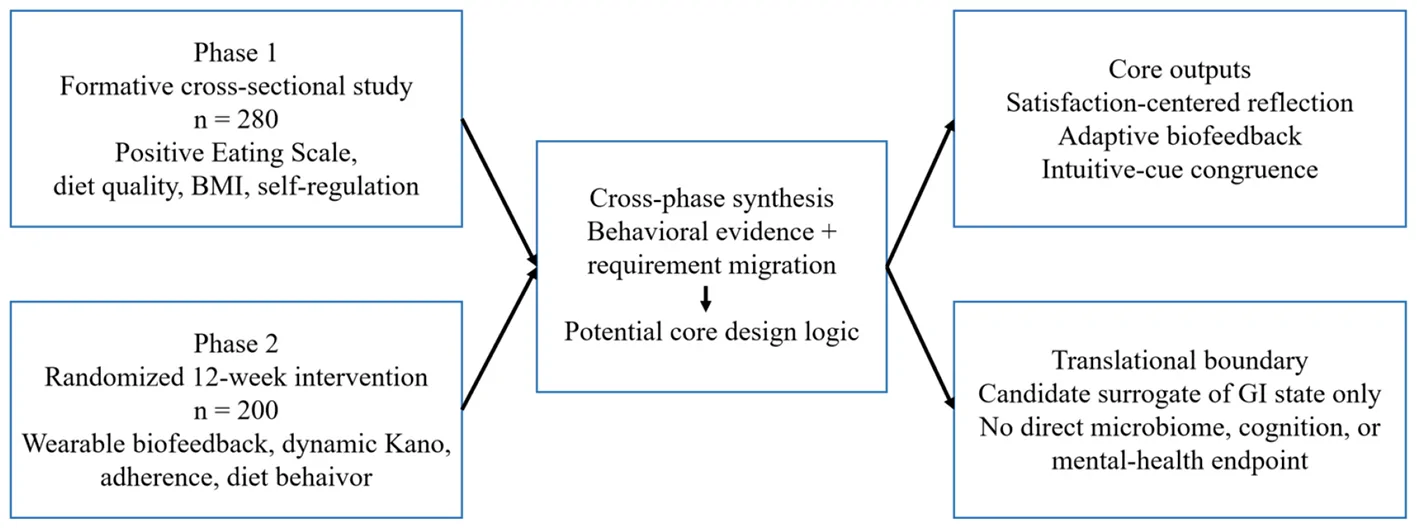
Integrated logic of the design as a two-phase evaluation. This study is designed as a two-phase evaluation: a formative behavioral phase, a longitudinal intervention phase, and a cross-phase synthesis that yields candidate design requirements while explicitly distinguishing present findings from downstream gut–brain-axis efficacy claims.

In light of the aforementioned background, the present paper reports a two-phase research program designed to answer a narrower, more defensible translational question: namely, what behavioral and HCI architecture should underlie an abdominal sound–enabled dietary self-regulation system for Taiwanese university students? Phase 1 of the study identified candidate behavioral targets using positive-eating data. Phase 2 examined the temporal migration of wearable HCI requirements during a 12-week intervention. The following hypothesis was formulated: it is hypothesized that satisfaction with eating would demonstrate a more favorable behavioral and weight-related profile than pleasure when eating. Furthermore, it is predicted that wearable features would undergo dynamic Kano migration over time. Finally, it is anticipated that cross-phase synthesis would converge on a small set of core requirements for a future abdominal sound–enabled system. The integrated two-phase design and cross-phase synthesis are summarized in Figure 1.

### 1.1 Related work: abdominal acoustic sensing, gut-brain-axis plausibility, and HCI translation

In the context of gut physiology, gastrointestinal activity generates acoustically discernible patterns that may be relevant to internal fuel-state dynamics. Bowel sounds (BS) are the result of normal physiological activity and have a long history of clinical use for evaluating gut motility and identifying problems such as bowel obstruction, ileus, or gastrointestinal bleeding (29, 30). Recent studies have revived efforts to quantify bowel sounds using digital sensors, signal-processing methods, and artificial intelligence algorithms (30, 31). Digital stethoscopes and wearable acoustic sensors have been demonstrated to facilitate continuous, high-resolution recording of bowel sounds in real-world settings (32–34). In the present manuscript, abdominal sounds are considered a candidate timing and context signal for ingestive behavior rather than a stand-alone disease biomarker (35–38).

This approach is appealing because it addresses a crucial yet often overlooked aspect of behavior-change systems: the restoration of bodily sources of information (39). The study reveals significant disparities in gastric motility, gut hormone release, and the interactions between gut hormones and motility and appetite parameters between healthy individuals and those with obesity (40, 41). The psychobiological background is equally important. It is important to note that diet does not influence behavior in isolation; rather, it operates on the gut and brain through sensory afferents, vagal signaling, microbiota-related pathways, inflammatory processes, and reward-control circuitry. These factors are increasingly defining the gut-brain axis literature (42, 43).

Precision Nutrition (PN) has demonstrated that glycemic responses, metabolic reactions, and dietary effects vary substantially between people (44, 45). Nevertheless, there is a dearth of literature that systematically translates this complexity into behaviorally usable support. The capacity of PN to enable the identification of a response to the query, “What is the most beneficial dietary regime for optimal health and wellbeing?” is predicated on the understanding that the health implications and responses to dietary modification are subject to temporal fluctuations (46, 47). In most implementations, precision is concentrated in backend analytics rather than distributed evenly across the moment-to-moment user experience. The Interoceptive Precision Nutrition Interface (IPNI) concept integrates the gut-brain-HCI circuitry for hunger perception and self-regulated intake control, and reframes abdominal acoustic sensing as a temporal glue: it is not the entire precision-nutrition system, but it can connect biological individuality to lived dietary timing, satiety congruence, and adaptive prompting. In this regard, IPNI facilitates translating precision nutrition principles into practical, implementable strategies at the human interface.1

**Figure 2:**
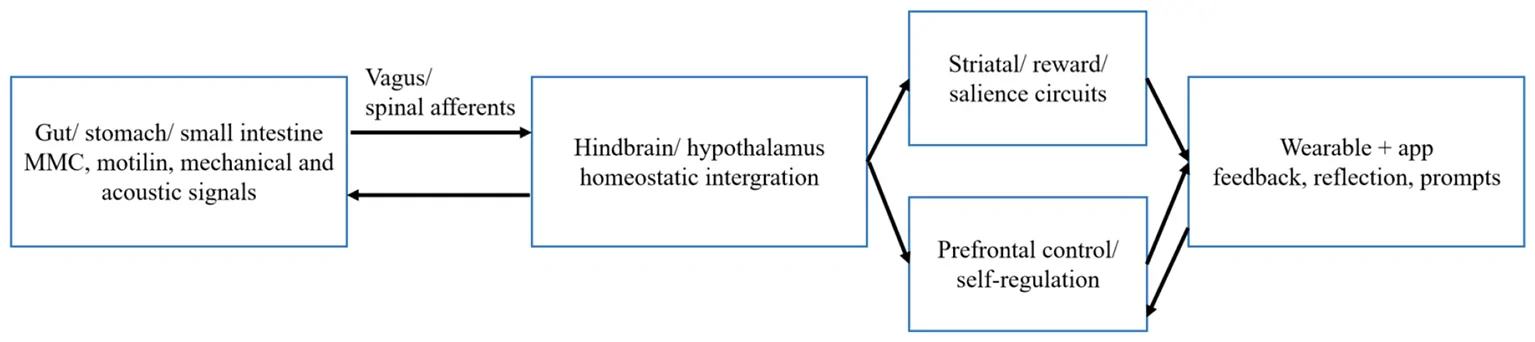
The Interoceptive Precision Nutrition Interface (IPNI) concept: the gut-brain-HCI circuitry for hunger perception and self-regulation intake control.

The present concept does not reduce itself to those mechanisms, but it is designed to make them self-regulation behaviorally approachable in everyday settings (Figure 2).

## 2 Materials and methods

### 2.1 Overall research design

The present study comprises two empirically linked phases: a formative, cross-sectional behavioral phase and a randomized, 12-week formative intervention phase. The overall research design is outlined in Figure 1. The objective of the study was not to pool participants across phases or to test a gut-brain-axis mechanism. Conversely, the integrated analysis derived candidate behavioral and HCI requirements from phase-specific evidence. Phase 1 identified positive eating constructs associated with dietary behavior and BMI. Phase 2 of the study described how requirements for wearable HCI changed during use. The phase-specific designs, sample characteristics, key variables, and primary analytic roles are summarized in Table 1.

**Table 1:** Study design, sample characteristics, and analytic role.

Phase	Design	Sample	Key variables	Primary analytic role
Phase 1	Cross-sectional formative study	*n* = 280; 45% male; mean age 22 ± 4 years	Positive Eating Scale, restrained/emotional eating, regulation strategies, intuitive eating, diet quality, BMI	Identify behavioral targets for a wearable dietary self-regulation system.
Phase 2	Randomized 12-week intervention	*n* = 200; intervention *n* = 100; control *n* = 100; 62% female; mean age 22.1 ± 4.1 years	Kano attributes, CS/DS coefficients, adherence, fruit-and-vegetable intake, unhealthy snack frequency, and user satisfaction	Identify time-varying HCI requirements and outcome-relevant features

The cross-phase synthesis was conducted as a design-level translation rather than a participant-level pooled modeling approach. A requirement was designated as a candidate core requirement when Phase 1 supported its behavioral relevance and Phase 2 suggested that the corresponding HCI attribute was essential or becoming essential. A requirement was designated as ‘cautionary' when it demonstrated short-term appeal but equivocal or potentially unfavorable dietary or weight-related associations.

### 2.2 Phase 1: formative cross-sectional study

The initial phase of the study included 280 Taiwanese university students, of whom 45% were male and had a mean age of 22 years (±4 years). The participants completed a paper-based survey on food choice, eating behavior, and health-related perceptions. Exclusion criteria included incomplete age or sex information and subjects who failed to complete at least half of the questionnaire. This study has received ethical review approval.

The primary measure employed was the 8-item Positive Eating Scale, which encompasses Satisfaction with Eating (items 1–4) and Pleasure when Eating (items 5–8) (12). The additional variables include maladaptive eating (restrained and emotional eating), self-regulatory strategies (portion-size and calorie-focused regulation), adaptive internal regulation (intuitive eating), health-related orientation (health consciousness and perceived health), diet-related behavior (food-group intake and diet quality index), and BMI.

Phase 1 analyses examined psychometric structure using principal component analysis with ProMax rotation, sampling adequacy, Bartlett's test of sphericity, and Cronbach's alpha. The present study evaluated sex differences, Pearson correlations, and hierarchical regression models for diet quality and BMI using the phase-specific results available to the present integrated report.

### 2.3 Phase 2: randomized formative 12-week intervention

In the second phase of the study, a randomized, formative intervention was conducted over 12 weeks. This phase of the study involved 200 Taiwanese university students, with a mean age of 22.1 years (±4.1 years) and 62% female. Participants were randomly assigned to either an intervention group (*n* = 100) or a control group (*n* = 100). The inclusion criteria encompassed the following: at least one unhealthy snack episode per day; a daily intake of fruit and vegetables below 5 portions; the absence of a chronic dietary disorder; and the non-use of a dietary management wearable. The intervention lasted 12 weeks.

The control group received conventional dietary education. The intervention group used a wearable biofeedback system that combined state-related sensing, behavioral tracking, and adaptive HCI features. The study was approved by the Institutional Review Board (IRB No.: KY2021-609).

Twelve HCI attributes were evaluated using Kano analysis, namely: real-time biofeedback visualization, personalized goal notifications, short-term outcome feedback, social sharing, adaptive notification frequency, simplified data dashboard, reward system, customizable alerts, long-term progress reports, voice-guided feedback, environmental cue mapping, and battery life indicator. The assessment encompassed the evaluation of pre- and post-intervention Kano categories, Better coefficients (CS), Worse coefficients (DS), and feature-trajectory shifts. The intervention also reported fruit and vegetable intake, unhealthy snack frequency, device adherence, and user satisfaction.

### 2.4 Potential abdominal sound-enabled system architecture

This section describes the potential architecture of a system that could detect and process abdominal sounds. The evaluation of the intervention focused on the behavioral and interface layers. The present manuscript, therefore, treats abdominal sounds as a candidate physiological timing/context input for a future system, rather than as evidence of validated gut-state classification in the current sample.

Within the proposed architecture, abdominal sounds are positioned only as a possible timing and context-awareness layer. The potential benefits of such an approach include identifying candidate meal-state windows, postprandial phases, gastrointestinal quiescence or activity, and context-sensitive moments for feedback delivery. Nevertheless, these measures do not substitute for established biological metrics or provide validation for gut-brain-axis mechanisms. This framing is consistent with prior bowel-sound literature and remains cautious about what acoustic signals can and cannot infer (25–29, 35–38).

### 2.5 Statistical approach

The results of Phase 1 psychometric analysis were summarized using the following methods: KMO, Bartlett's test, factor loadings, and reliability coefficients. The associations between positive-eating subscales and behavioral or health variables were summarized using Pearson correlation coefficients. Hierarchical regressions reported unstandardized coefficients, standard errors, and, when available, standardized beta weights in the created analysis.

Phase 2 of the project provided a descriptive summary of the Kano migration. The multinomial logistic model reported provided information on model fit (chi-square = 47.32, *p* < 0.001; pseudo-R2 = 0.12), thereby establishing a correlation between baseline CS/DS coefficients and subsequent trajectory shifts. For group-level outcomes, mean differences and 95% confidence intervals were retained, and standardized mean differences were calculated from the reported means and standard deviations, with *n* = 100 per group. Due to the unavailability of participant-level data, incorporating an adjusted efficacy model, an imputation model, or a sensitivity analysis was not feasible. The interpretation of self-reported dietary measures in both phases was undertaken with caution, given the recognized limitations inherent to nutritional epidemiology and to the plausibility of self-reported dietary data (48–50).

## 3 Results

### 3.1 Phase 1 psychometric performance

**Figure 3:**
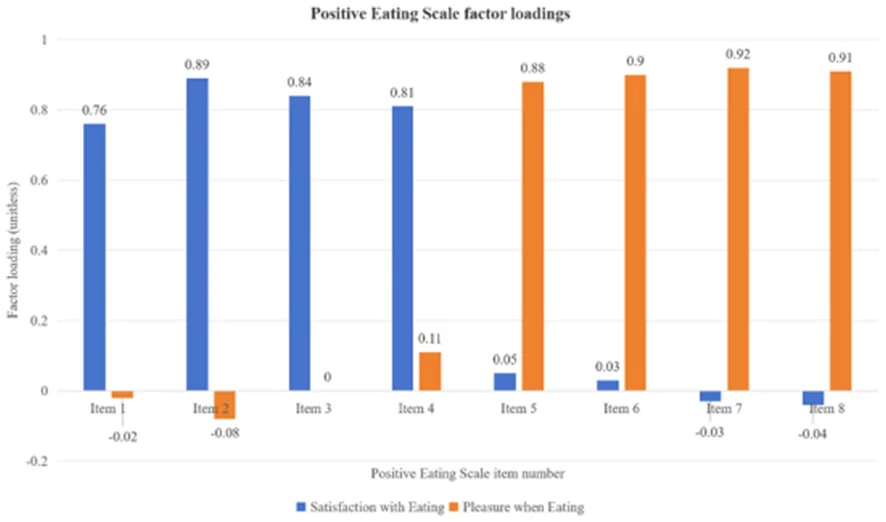
Positive eating scale factor loadings. Items 1–4 loaded primarily on Satisfaction with Eating, whereas items 5–8 loaded primarily on Pleasure when Eating, thereby supporting the two-factor interpretation employed in this study.

The Positive Eating Scale yielded a clear two-factor structure in the Taiwanese sample. The adequacy of the samples was strong (KMO = 0.85), Bartlett's test was highly significant (χ^2^(28) = 14,605.46, *p* < 0.001), and the item-loading pattern aligned with the expected distinction between satisfaction with eating and pleasure when eating. The internal consistency of the scale was satisfactory for satisfaction (α = 0.85), pleasure (α = 0.92), and the total scale (α = 0.87) (see Table 2, Figure 3).

**Table 2:** Positive Eating Scale item loadings in the Taiwanese sample.

Item	Statement	Satisfaction loading	Pleasure loading
1	I eat in a way that makes me feel good.	0.76	−0.02
2	Overall, I am satisfied with my eating behavior.	0.89	−0.08
3	I am relaxed about eating.	0.84	0.00
4	I have a good relationship with eating.	0.81	0.11
5	Eating is a pleasure for me.	0.05	0.88
6	I enjoy eating.	0.03	0.90
7	Eating is fun for me.	−0.03	0.92
8	Eating is something nice for me.	−0.04	0.91

The KMO measure was 0.85, and Bartlett's test of sphericity was significant (χ^2^(28) = 14,605.46, *p* < 0.001). Internal consistency was satisfactory for Satisfaction with Eating (α = 0.85), Pleasure when Eating (α = 0.92), and the total scale (α = 0.87).

The mean scores on the Total Positive Eating Scale were comparable between men and women; however, the two subscales diverged in theoretically meaningful ways. The results indicated that male participants exhibited marginally higher satisfaction with eating (3.4 ± 0.5 vs. 3.3 ± 0.6, *p* < 0.01), while female participants demonstrated slightly higher pleasure associated with eating (3.47 ± 0.62 vs. 3.54 ± 0.60, *p* < 0.01). The subgroup patterns that emerged provided a rationale for maintaining the satisfaction and pleasure subscales as discrete entities within the integrated design analysis framework.

### 3.2 Satisfaction with eating showed a stronger behavioral signal

Across the entire sample, satisfaction with eating showed a distinctly more favorable pattern than pleasure. The study found a negative correlation between satisfaction and restrained eating (*r* = −0.38) and emotional eating (*r* = −0.20). In contrast, positive associations were observed between satisfaction and portion-size regulation (*r* = 0.17) and negative associations with calorie-focused regulation (*r* = −0.10). Furthermore, a moderate correlation was observed between the variables under investigation and intuitive eating (*r* = 0.49), health consciousness (*r* = 0.18), and perceived health (*r* = 0.30) (see Table 3).

**Table 3:** Selected correlations linking positive eating to behavioral and health-related variables.

Variable	Satisfaction (overall r)	Pleasure (overall r)	Notable subgroup pattern
Restrained eating	−0.38^***^	−0.06^**^	Women: stronger inverse association with satisfaction (*r* = −0.45)
Emotional eating	−0.20^***^	0.09^***^	Men: pleasure positively associated (*r* = 0.14)
Portion-size regulation	0.17^***^	−0.07^***^	Women: stronger positive association for satisfaction (*r* = 0.25)
Calorie-focused regulation	−0.10^***^	−0.05	Negative association with satisfaction in both sexes
Intuitive eating	0.49^***^	0.17^***^	Women: stronger satisfaction association (*r* = 0.54)
Perceived health	0.30^***^	0.17^***^	Consistent positive association in both sexes
Diet quality index	0.09^***^	0.03	Women: satisfaction *r* = 0.15; pleasure near null
BMI (kg/m^2^)	−0.25^***^	−0.06^**^	Men: satisfaction *r* = −0.29; pleasure near null in zero-order analysis

^**^p < 0.01; ^***^p < 0.001.

In contrast, the phenomenon of pleasure exhibited a weaker and more varied profile. The present study found a positive relationship between pleasure and both intuitive eating (*r* = 0.17) and perceived health (*r* = 0.17). However, the data also demonstrated a positive association between pleasure and emotional eating (*r* = 0.09 overall; *r* = 0.14 in men), and a negligible relationship with diet quality. The results of the study demonstrated a modest positive association between satisfaction and the consumption of vegetables, fruits, whole grains, and diet quality, alongside a negative association with BMI (*r* = −0.25 overall; *r* = −0.29 in men) (Table 3).

The regression models further substantiated this distinction. In women, satisfaction independently predicted better diet quality (*b* = 0.30, SE = 0.07, β = 0.16, *p* < 0.001), whereas pleasure did not. In the context of BMI models, satisfaction has been shown to independently predict lower BMI in both men (β = −0.19, *p* < 0.001) and women (β = −0.17, *p* < 0.001). The results of the study indicated a positive correlation between BMI and pleasure in male subjects (β = 0.10, *p* < 0.01, *b* = 0.58, SE = 0.18). In contrast, a smaller yet non-significant positive association was observed in female subjects (Table 4). Collectively, these findings indicated that satisfaction with eating – and not merely pleasure – is the most defensible behavioral anchor for the wearable system.

**Table 4:** Standardized beta weights from hierarchical regression models.

Predictor	Diet quality: men β	Diet quality: women β	BMI: men β	BMI: women β
Age	−0.01	−0.01	0.13^***^	0.12^***^
Restrained eating	0.05	0.07	0.08	0.11^**^
Emotional eating	−0.08	−0.10^**^	−0.01	−0.06
Intuitive eating	−0.07	−0.08	−0.19^***^	−0.18^***^
Health consciousness	0.22^***^	0.24^***^	−0.17^***^	−0.12^***^
PES satisfaction	0.05	0.16^***^	−0.19^***^	−0.17^***^
PES pleasure	0.01	−0.06	0.10^**^	0.08

Women: satisfaction independently predicted better diet quality. Men and women: satisfaction independently predicted lower BMI. Men's BMI model showed a positive association with pleasure. ^**^p < 0.01; ^***^p < 0.001.

### 3.3 Phase 2 dynamic Kano migration

**Figure 4:**
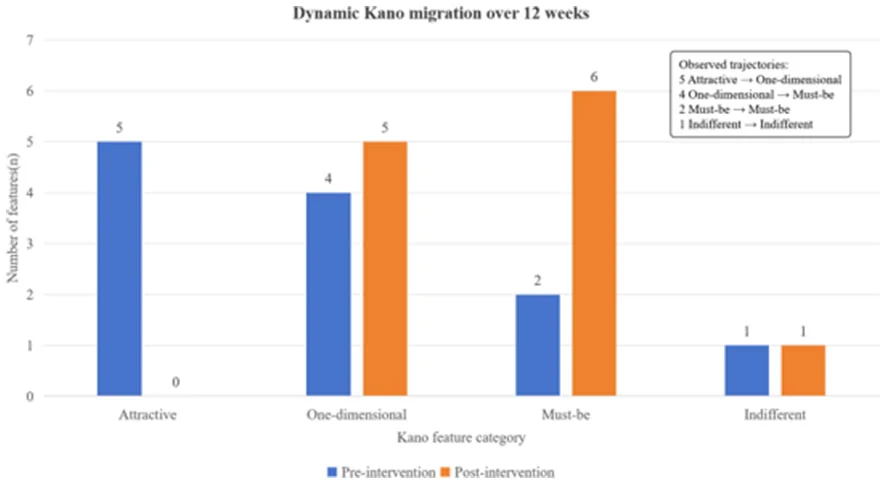
Dynamic Kano migration over 12 weeks. Over time, attractive features decreased in number, one-dimensional features increased modestly, and must-be features increased sharply. This indicates that long-term value depended on the maturation of core functions rather than on novelty alone.

The pre-intervention Kano profile comprised five attractive features, four one-dimensional features, two must-be features, and one indifferent feature. Over a period of 12 weeks, all five attractive features migrated to one-dimensional qualities, four one-dimensional features migrated to must-be qualities, and the two must-be features and the indifferent feature remained stable (see Table 5 and Figure 4).

**Table 5:** Dynamic Kano classification of wearable HCI attributes.

HCI feature	Pre	CS	DS	Post	Trajectory
Real-time biofeedback visualization	One-dimensional	0.62	0.68	Must-be	O → M
Personalized goal notifications	One-dimensional	0.59	0.70	Must-be	O → M
Short-term outcome feedback	Attractive	0.67	0.35	One-dimensional	A → O
Social sharing	Attractive	0.71	0.29	One-dimensional	A → O
Adaptive notification frequency	Attractive	0.69	0.32	One-dimensional	A → O
Simplified data dashboard	Must-be	0.38	0.72	Must-be	M → M
Reward system	Attractive	0.73	0.27	One-dimensional	A → O
Customizable alerts	One-dimensional	0.61	0.65	Must-be	O → M
Long-term progress reports	Indifferent	0.31	0.25	Indifferent	I → I
Voice-guided feedback	Attractive	0.68	0.30	One-dimensional	A → O
Environmental cue mapping	One-dimensional	0.58	0.63	Must-be	O → M
Battery life indicator	Must-be	0.36	0.75	Must-be	M → M

A, attractive; O, one-dimensional; M, must-be; I, indifferent; CS, better coefficient; DS, worse coefficient; trajectory notation such as A -> O indicates migration from the pre-intervention Kano category to the post-intervention Kano category.

The multinomial logistic model demonstrated that these trajectories were not random. The baseline coefficient structure was a reliable predictor of subsequent migration (χ^2^ = 47.32, *p* < 0.001; pseudo-*R*^2^ = 0.12). It was predicted that higher initial CS values would indicate attractive-to-one-dimensional transitions (β = 0.83, *p* < 0.001). In contrast, higher initial DS values would be indicative of one-dimensional-to-must-be transitions (β = 0.76, *p* < 0.001). This pattern suggests that novelty-driven features may enhance early appeal, whereas dissatisfaction-sensitive features are more likely to become everyday expectations.

### 3.4 Intervention outcomes

The intervention outcomes are compared with those of the control group. Intervention participants reported higher consumption of fruit and vegetables (4.2 ± 1.3 portions/day compared with 2.8 ± 1.1 portions/day), lower frequency of unhealthy snacks (0.9 ± 0.6 times/day compared with 1.7 ± 0.8 times/day), higher adherence to the device (6.2 ± 1.8 h/day compared with 0.3 ± 0.5 h/day), and higher user satisfaction (5.8 ± 1.1 compared with 3.2 ± 1.3). As shown in Table 6, all group differences were significant (*p* < 0.001). It is important to note that these results are descriptive and represent group-level outcomes rather than adjusted estimates of clinical efficacy.

**Table 6:** Group-level outcomes in the 12-week intervention.

Outcome	Intervention (*n* = 100)	Control (*n* = 100)	Mean difference (95% CI) standardized mean difference *d*	*p*
Fruit-and-vegetable intake (portions/day)	4.2 ± 1.3	2.8 ± 1.1	1.4 (1.1 to 1.7); *d* = 1.16	< 0.001
Unhealthy snack frequency (times/day)	0.9 ± 0.6	1.7 ± 0.8	−0.8 (−1.0–−0.6); *d* = −1.13	< 0.001
Device adherence (hours/day)	6.2 ± 1.8	0.3 ± 0.5	5.9 (5.6–6.2); *d* = 4.47	< 0.001
User satisfaction (7-point scale)	5.8 ± 1.1	3.2 ± 1.3	2.6 (2.3–2.9); *d* = 2.16	< 0.001

The reported correlations between alignment with dynamic Kano trajectories and adherence (*r* = 0.64, *p* < 0.001) and fruit-and-vegetable intake (*r* = 0.58, *p* < 0.001) are interpreted as design-relevant associations. The findings of this study lend support to the notion that it is advantageous for designers to create features that users come to expect rather than rely exclusively on those that are initially attractive.

### 3.5 Cross-phase synthesis

**Figure 5:**
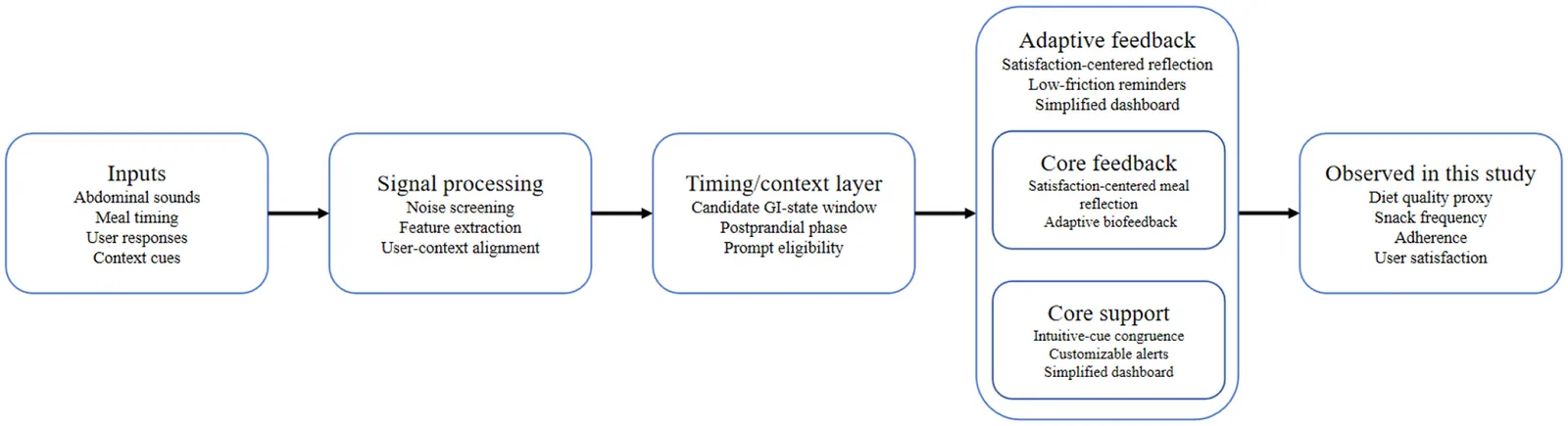
Abdominal-sound-enabled feedback workflow and validation boundary. Abdominal sounds are positioned as a candidate non-invasive timing/context signal for adaptive feedback. The validation of acoustic classifier performance and gut-brain-axis endpoints is required for future studies.

The cross-phase synthesis was discussed. It was noted that this process had resulted in a candidate design specification for a future abdominal-sound-enabled dietary self-regulation system. This information is presented in Table 7 and Figure 5. This synthesis translates phase-specific evidence into design requirements; it does not pool participants across phases or prove a physiological mechanism. Four requirements were identified as central to the program: satisfaction-centered, non-punitive meal reflection; intuitive-cue congruence rather than calorie fixation; adaptive timing for biofeedback and notifications; and simplified, low-friction interaction.

**Table 7:** Evidence-based design translation matrix for a future abdominal sound–enabled system.

Candidate requirement	Supporting evidence	Design implication	Abdominal sound–enabled implementation	Priority
Satisfaction-centered meal reflection	Satisfaction correlated with restrained eating (*r* = −0.38), intuitive eating (*r* = 0.49), perceived health (*r* = 0.30), and BMI (*r* = −0.25)	Use non-judgmental post-meal reflection as a default interaction	Trigger meal reflection during postprandial windows rather than after arbitrary clock-based intervals	Core
Intuitive-cue congruence prompts	Satisfaction aligned with intuitive eating and opposed calorie-focused control	Ask about hunger, satiety, and meal adequacy before prescribing restriction.	Use candidate GI-state cues to time prompts about satiety, comfort, and congruence.	Core
Adaptive biofeedback and reminder timing	Biofeedback, personalized notifications, and customizable alerts became must-have features.	Treat adaptive timing and low-friction reminders as expected functionality.	Adjust prompt frequency to response history and likely meal-state windows	Core
Simplified dashboard	Dashboard remained must-be across the full intervention	Keep key metrics visible, concise, and low burden	Display only essential summaries linked to the current state and recent behavior	Core
Pleasure-preserving reinforcement	Pleasure improved affective appeal but did not improve diet quality; men's BMI β = 0.10	Use celebratory feedback sparingly and only with regulatory safeguards	Allow positive reinforcement after satiety-congruent, non-impulsive episodes	Secondary / guarded
Calorie-centric surveillance	Satisfaction was negatively associated with calorie-focused regulation	Avoid default calorie salience in the core workflow	Make calorie views optional or clinician-enabled rather than always visible	Cautionary

Pleasure-oriented reinforcement was retained solely as a secondary guarded enhancer. While the results suggest that pleasure may support engagement, the available Phase 1 results do not demonstrate a correlation with superior diet quality. Furthermore, a positive association between pleasure and BMI was observed among male participants. Consequently, hedonic reinforcement should be constrained by satisfaction, satiety congruence, and a non-impulsive eating context.

## 4 Discussion

### 4.1 Main interpretation

The principal contribution of this study identifies behavioral and HCI conditions under which a future abdominal-sound-enabled dietary self-regulation system could be scientifically testable and acceptable to Taiwanese university students. Firstly, observed behavioral associations from Phase 1; secondly, observed HCI requirement migration and descriptive outcomes from Phase 2; and thirdly, a future system architecture that would necessitate direct acoustic, microbiome, metabolomic, inflammatory, mood, cognitive, and clinical validation before mechanistic claims could be substantiated.

The present findings delineate the behavioral and HCI conditions under which an abdominal sound–enabled nutrition system might become scientifically and practically credible. Across both phases, the convergent signal favored a satisfaction-centered, low-friction, adaptive interface over a calorie-dominant or pleasure-maximizing logic.

This distinction is significant because dietary technologies frequently equate engagement with effectiveness. The Phase 1 results suggest that this simplification may be inaccurate. Satisfaction with eating was found to serve as a broader evaluative marker of workable, acceptable, and bodily-congruent eating. In contrast, the concept of pleasure captured a more immediate hedonic dimension that did not reliably translate into better diet quality or lower BMI. From a system-design perspective, the concept of delight is useful; however, it is not a substitute for regulatory fit.

### 4.2 What dynamic Kano adds

Phase 2 extends this argument from content to time. The dynamic Kano analysis showed that appealing characteristics, such as social sharing, rewards, and voice-guided prompts, did not persist once students became familiar with the system. Conversely, real-time biofeedback, personalized notifications, customizable alerts, and environmental cue mapping have become progressively essential. This is precisely the type of temporal shift in requirements that many digital nutrition tools fail to anticipate.

The practical implication is that long-term adherence is less dependent on preserving novelty and more on anticipating what will become non-negotiable. Consequently, a wearable system for diet-related self-regulation should be designed around features that evolve into expectations, rather than those that demonstrate efficacy in a preliminary usability test.

### 4.3 Relevance to gut–brain-axis research

This two-phase study did not directly measure abdominal acoustics or gut–brain-axis mechanisms. Its contribution is upstream and translational: it specifies behavioral and HCI requirements for a future abdominal-sound-enabled system and delineates the acoustic, physiological, cognitive, and mental-health endpoints that require prospective validation.

The subject's research topic focuses on human studies of dietary modulation of the gut–brain axis and its cognitive and mental health consequences. Consequently, the present study should be interpreted as an upstream translational contribution rather than a direct efficacy study.

Abdominal sounds have been identified as a potential indicator of the timing of daily interventions, offering a practical solution for patient management. However, the current evidence base does not support the hypothesis that abdominal acoustics can be considered a direct proxy for microbiota composition, neural activity, cognition, or mood. At present, their value lies in their potential to serve as surrogate markers of gastrointestinal state, with the possibility of future integration with stool, metabolomic, inflammatory, mood, and cognitive endpoints. The work is pertinent because it addresses a significant bottleneck in gut–brain research: translating biologically plausible yet intermittently measured signals into ecologically valid and user-acceptable support for interventions. In the future, this “eating behavior monitoring (EBM) device system” will explore behavioral/pharmacological strategies in eating disorders and weight management with the potential to control food intake.

### 4.4 Implications for Taiwanese university students

The system logic identified here is particularly relevant to Taiwanese university students, whose eating behavior often unfolds in constrained and unstable contexts. In such a population, the impediment to healthier eating is frequently not nutritional ignorance alone, but rather the absence of a viable day-to-day regulatory framework. A wearable interface that queries whether a meal was both sufficient and acceptable, adapts timing of reminders, and eschews punitive calorie salience is likely to be more acceptable than a tool centered on counting and issuing warnings.

The findings, which are specific to the sexes, also suggest that personalization rules may be warranted. The utility of diet quality for satisfaction was more clearly demonstrated in women, whereas the BMI-risk signal was more clearly demonstrated in men. It is important to note that these subgroup patterns are not deterministic; however, they do provide a rationale for considering sex as one of several moderators in future onboarding and personalization logic.

### 4.5 Strengths and limitations

The present study is notable for three principal strengths. Firstly, it combines behavioral and user-requirement evidence rather than treating sensing and interface design as discrete problems. Secondly, it employs a real-world university context and a 12-week intervention horizon, both of which offer greater insight than laboratory-only usability tests. Thirdly, it offers a restrained interpretation of abdominal sounds as a candidate translational signal, rather than an overextended biomarker claim.

It is imperative to acknowledge the significance of several limitations. The study did not encompass the measurement of direct microbiome, inflammatory, metabolomic, cognitive, and mental health outcomes. The predominant theoretical framework perceives eating behavior as a synthesis of involuntary and deliberate mechanisms. Physiological theories have emphasized the influence of biological signals on behavior. However, they have been criticized for failing to explain how key non-physiological factors (such as environmental cues and emotions) drive eating. Moreover, emerging integrative frameworks, such as “uncontrolled eating,” although grounded in eating behavior data, lack a solid foundation in extant theories of eating behavior.

The study integrated phase-specific findings rather than performing a pooled participant-level analysis across phases. The reliability of dietary outcomes depends on self-report, a method susceptible to reporting error (48–50). Finally, the university student sample enhances contextual relevance but limits generalizability to older or clinical populations.

### 4.6 Future research

The subsequent study should be a prospectively registered, randomized trial that tests the hypothesis that a satisfaction-centered, dynamically adaptive, abdominal-sound-enabled platform improves dietary self-regulation compared with a standard calorie-centered wearable.

The subsequent protocol must encompass the endpoints not present in this instance, namely: labeled abdominal-acoustic recordings, prespecified classifier validation, stool microbiome profiles, selected microbial metabolites, inflammatory markers, mood and stress measures, gastrointestinal symptom reports, and cognitive performance outcomes.

## 5 Conclusion

The findings of this two-phase study lend support to a design conclusion: if a future abdominal-sound-enabled dietary wearable is to be behaviorally credible, its core logic should be satisfaction-centered, adaptive, and low in punitive friction. Satisfaction with eating demonstrated the broadest favorable behavioral and weight-related associations, whereas pleasure alone was insufficient as a guide to healthier dietary regulation.

The intervention phase indicated that wearable value is dynamic: real-time biofeedback, personalized prompting, customizable alerts, and a simplified dashboard became expected rather than optional features. Consequently, the development of future abdominal-sound-enabled systems should prioritize treating acoustic input as a candidate timing/context layer to facilitate nonjudgmental meal reflection, adaptive timing, and constrained hedonic reinforcement.

## Data Availability

The original contributions presented in the study are included in the article. Further inquiries can be directed to the corresponding author.
